# Impact of extra-anatomical bypass on coarctation fluid dynamics using patient-specific lumped parameter and Lattice Boltzmann modeling

**DOI:** 10.1038/s41598-022-12894-y

**Published:** 2022-06-11

**Authors:** Reza Sadeghi, Benjamin Tomka, Seyedvahid Khodaei, MohammadAli Daeian, Krishna Gandhi, Julio Garcia, Zahra Keshavarz-Motamed

**Affiliations:** 1 Department of Mechanical Engineering, McMaster University, Hamilton, Canada ON; 2 Stephenson Cardiac Imaging Centre, Libin Cardiovascular Institute of Alberta, Calgary, AB Canada; 3 Department of Radiology, University of Calgary, Calgary, AB Canada; 4 Department of Cardiac Sciences, University of Calgary, Calgary, AB Canada; 5 Alberta Children’s Hospital Research Institute, Calgary, AB Canada; 6 School of Biomedical Engineering, McMaster University, Hamilton, ON Canada; 7 School of Computational Science and Engineering, McMaster University, Hamilton, ON Canada

**Keywords:** Engineering, Biomedical engineering, Cardiology, Interventional cardiology

## Abstract

Accurate hemodynamic analysis is not only crucial for successful diagnosis of coarctation of the aorta (COA), but intervention decisions also rely on the hemodynamics assessment in both pre and post intervention states to minimize patient risks. Despite ongoing advances in surgical techniques for COA treatments, the impacts of extra-anatomic bypass grafting, a surgical technique to treat COA, on the aorta are not always benign. Our objective was to investigate the impact of bypass grafting on aortic hemodynamics. We investigated the impact of bypass grafting on aortic hemodynamics using a patient-specific computational-mechanics framework in three patients with COA who underwent bypass grafting. Our results describe that bypass grafting improved some hemodynamic metrics while worsened the others: (1) Doppler pressure gradient improved (decreased) in all patients; (2) Bypass graft did not reduce the flow rate substantially through the COA; (3) Systemic arterial compliance increased in patients #1 and 3 and didn’t change (improve) in patient 3; (4) Hypertension got worse in all patients; (5) The flow velocity magnitude improved (reduced) in patient 2 and 3 but did not improve significantly in patient 1; (6) There were elevated velocity magnitude, persistence of vortical flow structure, elevated turbulence characteristics, and elevated wall shear stress at the bypass graft junctions in all patients. We concluded that bypass graft may lead to pseudoaneurysm formation and potential aortic rupture as well as intimal hyperplasia due to the persistent abnormal and irregular aortic hemodynamics in some patients. Moreover, post-intervention, exposures of endothelial cells to high shear stress may lead to arterial remodeling, aneurysm, and rupture.

## Introduction

Coarctation of the aorta (COA) is a common congenital heart defect (CHD) which is recognized as a general arteriopathy involving a discrete stenosis or a longer, hypoplastic segment of the aortic isthmus^1,2^. COA is the 6th most prevalent CHD, occurring in 5–8% of all cases with an approximate incidence of 3/10,000 livebirths^1–6^. COA imposes significant afterload on the left ventricle (LV) which results in elevated wall stress, LV hypertrophy, LV dysfunction, the development of arterial collaterals, upper body hypertension, flow disturbance in the thoracic aorta, and decreased perfusion to the lower body^2,5^. Further impacts of COA include aortic dissection, aortic rupture, myocardial infarction, and heart failure^5,7^. If left untreated, COA carries dismal prognosis, several studies have shown an average survival age of 30–35 years, with a mortality rate of 75% by age 46^3,8,9^. The appropriate surgical technique for COA repair often remains unclear for adult patients^10^. Surgical techniques to treat COA include resection with end-to-end anastomosis, prosthetic patch aortoplasty, subclavian flap aortoplasty, interposition grafting and extra-anatomic bypass grafting^2,4,11,12^.

Extra-anatomical bypass grafting has been recommended for complex COA cases (COA coexists with the other valvular, vascular and ventricular diseases)^4,5,10,12–15^ as well as isolated COA cases (COA does not coexist with the other cardiovascular pathologies)^11,12^ in adults. Furthermore, surgical treatment for COA carries some risk for spinal cord injury, occurring in approximately 0.5% of patients being operated on for coarctation^16^, studies have shown that extra-anatomic bypass minimizes the risk of paraplegia^2,15^. When performing bypass grafting, proximally, the prosthetic conduit is anastomosed to the ascending aorta or the subclavian artery, distally the conduit is attached to the descending aorta^4,14,17^. This technique leaves the stenosed aorta in situ, but is able to provide adequate blood flow to the distal aorta^14^. While extra-anatomic bypass can be performed with low risk^15^, some adverse cases do exist and include prosthetic graft pseudoaneurysm, intimal hyperplasia and potential re-COA, all of which may require reoperation^18–24^.

The development and progression of cardiovascular diseases often may be explained by abnormal hemodynamics characterized by disturbed or turbulent flow, and adverse vascular biomechanics^25–31^, in addition to other clinical metrics (e.g., genetic predisposition, age, etc.). Blood flow often determines the form and function of the heart and surrounding vascular network and can dictate the functional and structural response of repair^29,31,32^. Blood flow analysis can be greatly useful for diagnostic and monitoring purposes as disturbed flow strongly influences vascular pathologies^27,32^. Moreover, there is currently no consensus on the ideal treatment method for isolated, complex, or recurrent COA^33,34^, and hemodynamics may provide insight to the optimal treatment method on a patient-specific basis.

A clinically useful tool, that can be used to accurately assess fluid dynamics in COA patients pre- and post- bypass grafting, should satisfy the following requirements: boundary conditions should be patient-specific and should be acquired non-invasively. Imposing patient-specific flow and pressure boundary conditions is critically important for a patient-specific computational diagnostic framework which should perform 3-D blood flow calculations. In addition, obtaining the required boundary conditions invasively (e.g., with cardiac catheterization) may put the patients at high risk which contradicts the whole purpose of the computational blood flow diagnostic framework. Therefore, the required boundary conditions should be acquired *non-invasively* in each individual patient.

There have been attempts for quantifying blood flow through bypass grafts using conventional macroscopic numerical methods based on the discretization of Navier–Stokes equations (finite difference method, finite volume method, finite element method, etc.)^18,19,35–39^. However, none of these models can satisfy the mentioned requirements as they do not have patient-specific boundary conditions and patient-specific geometry etc^18,19,35–39^.

*"Cardiology is flow”*^32^ and indeed one of the essential sources of cardiovascular mortality and morbidity can be explained on the basis of abnormal fluid dynamics, leading to the development and progression of cardiovascular disease^25,26,31,40,41^. To effectively evaluate risk status and create guidelines for intervention, precise quantification of a*ortic fluid dynamics*, is required. For this purpose and to satisfy the requirements described earlier, we developed a patient-specific computational-mechanics framework to investigate the impact of bypass grafting on aortic fluid dynamics in three patients with COA in both pre and post extra-anatomical bypass grafting conditions to provide novel analyses and interpretations of clinical data. To the best of our knowledge, this is the first study that investigates the effects of bypass grafting on COA hemodynamics.

## Materials and methods

**Figure 1:**
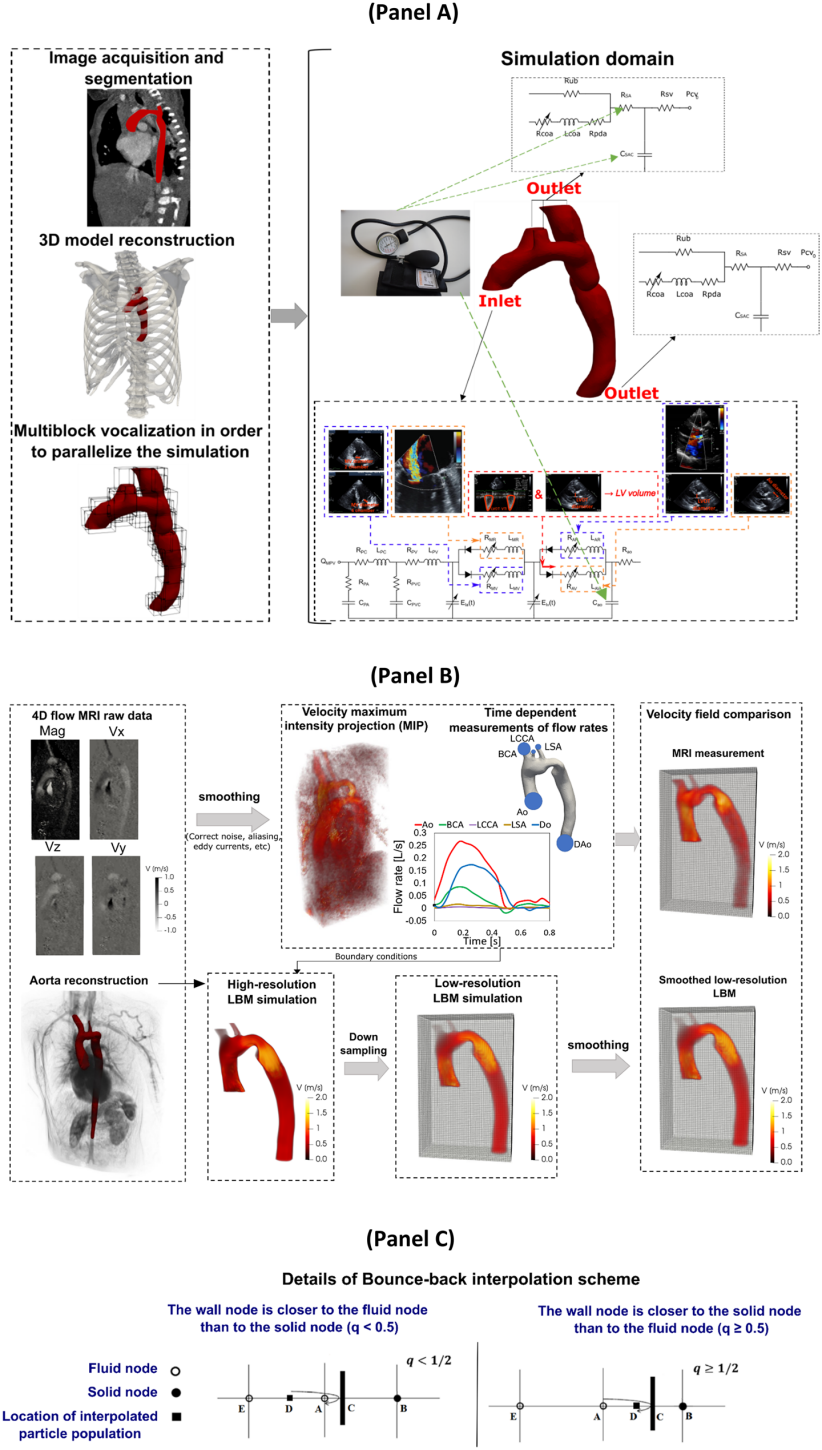
Reconstructed geometry and simulation domain. Schematic diagram of our developed patient-specific, image-based, computational-mechanics framework that dynamically couples the local hemodynamics with the global circulatory cardiovascular system to investigate the impact of COA and bypass grafting on fluid dynamics in these patients. We used CT images from patients to segment and reconstruct the 3D geometries of the complete aorta. These 3-D geometries were used for investigating hemodynamics using computational fluid dynamics. Local flow dynamics are greatly influenced by upstream and downstream flow conditions that are absent in the flow simulation domain. A lumped-parameter model simulates the function of the left side of the heart. Time-dependent inlet flow at ascending aorta and outlet pressure at descending aorta position were obtained from lumped parameter modeling and applied as boundary conditions. Boundary conditions of the aortic branches were adjusted to match the flow distribution; (**b**) We compared 4-D flow MRI data and results of our computational framework. The 3-D geometry of the complete aorta was reconstructed using MRI images and the entire volume of Down-sampled LBM data was smoothed (see *Four-dimensional flow magnetic resonance imaging (4-D flow MRI* section for more details); (**c**) Modeling complex geometries in LBM. Details of bounce-back interpolation scheme (Here A and E are fluid nodes, B is solid nodes and D represents the location of an interpolated population): (I) The wall-node ${\text{C}}$ is closer to the fluid-node ${\text{A}}$ than to the solid-node ${\text{B}}$ (${\text{q}} < 1/2$). In this case, interpolations are required to construct post collision state at node D. We constructed the unknown quantities at node A from particles population at node D that will travel to node A after bouncing back off the wall. (II) The wall-node $C$ is closer to the solid-node $B$ than to the fluid-node $A$ ($q \ge 1/2$). In this case, endpoint of propagation state (node D) lies between the boundary node (A) and the wall node (C) and the information of the particle leaving node A and arriving node D will be used to compute the unknown quantities at node A ^46,61,115^.

**Figure 2:**
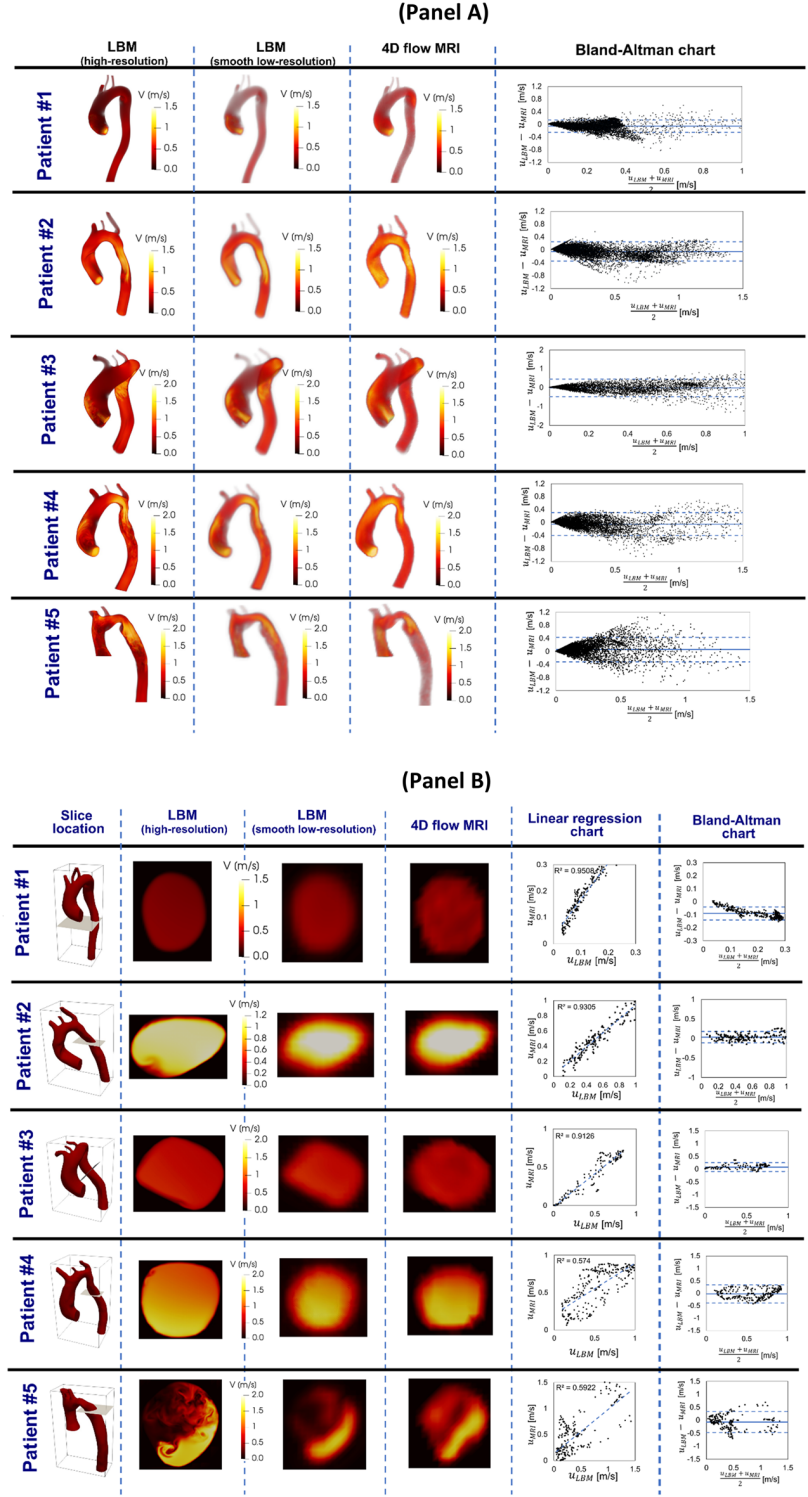
Validation against 4-D flow MRI. (**a**) We compared 4-D flow MRI data and results of the computational framework (based on lumped parameter model (LPM) and Lattice Boltzmann model (LBM)) in Patients #I to #V. (**a**) qualitatively (revealed in velocity mapping) and quantitatively by performing Pearson’s product moment correlation analysis on the entire domain at peak systole between smooth down-sampled LBM and 4D flow MRI measurements; (**b**) qualitatively (revealed in velocity mapping) and quantitatively by performing linear regression and Pearson’s product moment correlation analysis at sample cross sections at peak systole between smooth down-sampled LBM and PC-MRI measurements.

In this study, we simulated the 3-D blood flow dynamics in three patients with COA in pre- and post-intervention (extra-anatomical bypass graft) status using our developed computational fluid dynamics framework. This framework is based on our Doppler-based, patient-specific, lumped parameter modeling^29,42^ and 3-D lattice Boltzmann method (LBM; Smagorinsky subgrid scale model)^31^ implemented in the open-source OpenLB library^31^ (Fig. 1). Our Doppler-based, patient-specific lumped parameter model was further developed in this work to model complex coarctation (COA coexists with the other valvular, vascular and ventricular disease) and extra-anatomical bypass graft (Fig. 1, Table 1). The velocity fields calculated using Lattice Boltzmann model were validated against 4-D flow MRI measurements in five other COA patients (in addition to the three patients described above) (Figs. 1 and 2, see *Validation* section for all details).

**Table 1:** Summarized parameters used in the lumped parameter modeling to simulate all cases.

Description	Abbreviation	Value
**Valve parameters**		
Effective orifice area	EOA	Measured using DE
Inertance (mitral valve)	M_MV_	Constant value: 0.53 gcm^-2^
**Systematic circulation parameters**		
Aortic resistance	R_ao_	Constant value: 0.05 mmHg.s.mL^-1^
Aortic compliance	C_ao_	Initial value: 0.5 mL/mmHg
		Optimized based on brachial pressures
		*(Systolic and diastolic brachial pressures are optimization constraints)*
Systemic vein resistance	R_SV_	0.05 mmHg.s.mL^-1^
Systemic arteries and veins compliance	C_SAC_	Initial value: 2 mL/mmHg
		Optimized based on brachial pressures
		*(Systolic and diastolic brachial pressures are optimization constraints)*
systemic arteries resistance (including arteries, arterioles and capillaries)	R_SA_	Initial value: 0.8 mmHg.s.mL^-1^
		Optimized based on brachial pressures
		*(Systolic and diastolic brachial pressures are optimization constraints)*
Upper body resistance	R_ub_	*Modeling COA*: Initially adjusted to have 15% of total flow rate in healthy case when COA is not in the circuit
		*Modeling COA in presence of bypass graft*: adjusted to have total flow rate crossing aortic arch branches, calculated using Doppler echocardiography data
Proximal descending aorta resistance	R_pda_	Constant value: 0.05 mmHg.s.mL^-1^
Elastance Function*		
Maximum Elastance	E_max_	2.1 (LV)
		0.17 (LA)
Minimum Elastance	E_min_	0.06 (LV, LA)
Elastance ascending gradient	m_1_	1.32 (LV, LA)
Elastance descending gradient	m_2_	27.4 (LV)
		13.1 (LA)
Elastance ascending time translation	$\tau_{1}$	0.269 T (LV)
		0.110 T (LA)
Elastance descending time translation	$\tau_{2}$	0.452 T (LV)
		0.18 T (LA)
**Pulmonary circulation parameters**		
Pulmonary Vein Inertance	L_PV_	Constant value:0.0005 mmHg·s^2^·mL^-1^
Pulmonary Vein Resistance	R_PV_	Constant value: 0.002 mmHg·s·mL^-1^
Pulmonary Vein and capillary Resistance	R_PVC_	Constant value: 0.001 mmHg·s·mL^-1^
Pulmonary Vein and Capillary Compliance	C_PVC_	Constant value: 40 mL/mmHg
Pulmonary Capillary Inertance	L_PC_	Constant value: 0.0003 mmHg·s^2^·mL^-1^
Pulmonary Capillary Resistance	R_PC_	Constant value: 0.21 mmHg·s·mL^-1^
Pulmonary Arterial Resistance	R_PA_	Constant value: 0.01 mmHg·s·mL^-1^
Pulmonary Arterial Compliance	C_PA_	Constant value: 4 mL/mmHg
Mean Flow Rate of Pulmonary Valve	Q_MPV_	*Forward LVOT-SV* is the only input flow condition (measured using DE). *Q*_*MPV*_ *is a flow parameter that was optimized so that the lump-parameter model could reproduce the desirable DE-measured Forward LVOT-SV*
**Input flow condition**		
Forward left ventricular outflow tract stroke volume	Forward LVOT-SV	Measured using DE
**Output condition**		
Central venous pressure	P_CV0_	Constant value: 4 mmHg
**Other**		
Constant blood density	$\rho$	Constant value: 1050 kg/m^3^
Heart rate	HR	Measured using DE
Duration of cardiac cycle	T	Measured using DE
Systolic End Ejection time	T_EJ_	Measured using DE
End diastolic volume	EDV	Measured using DE
End systolic volume	ESV	Measured using DE

### Lattice Boltzmann method (LBM)

In a healthy vascular system, blood flow is mostly laminar, however, blood flow becomes distally turbulent in the presence of pathologies. The Reynolds-averaged Navier Stokes (RANS) methods are popular but have limited capability for adequate capturing of turbulence in pulsatile flows^43^. Direct numerical simulations (DNS) are very expensive computationally and are restricted to low Reynolds numbers. Large eddy simulation is a method between DNS and RANS and it was shown to be an appropriate choice for modelling pulsatile transitional and turbulence vascular flows^29^. In this study, we used a 3-D LBM-based computational fluid dynamics approach using LES (Smagorinsky subgrid scale model) to simulate blood flow through the vascular system.

All details about governing equations, Lattice Boltzmann method, single and multi -relaxation time (SRT and MRT) ^44,45^, turbulent modeling were presented elsewhere ^31^. Here, we provide a concise description as following:

#### Modeling surface curvature near the wall of complex geometries

An interpolated bounce-back scheme proposed by Bouzidi et al. ^46^was used in order to treat boundaries of inclined and complicated geometry. To evaluate the post-propagation state of fluid node A next to a curved solid wall, the distribution function (Fig. 1, panel C) used for this technique ^31^ was defined as follows:

1 $$f_{{\overline{\alpha }}} \left( {x_{A} ,{\text{t}} + \Delta {\text{t}}} \right) = \left\{ {\begin{array}{*{20}c} {\begin{array}{*{20}c} {2{\text{q}}f_{\alpha }^{c} \left( {x_{A} ,t} \right) - \left( {1 - 2{\text{q}}} \right)f_{\alpha }^{c} \left( {x_{E} ,t} \right)} & {{\text{q}} < \frac{1}{2}} \\ \end{array} } \\ {\begin{array}{*{20}c} {\frac{1}{{2{\text{q}}}}f_{\alpha }^{c} \left( {x_{A} ,{\text{t}}} \right) + \frac{{\left( {2{\text{q}} - 1} \right)}}{{2{\text{q}}}}f_{\alpha }^{c} \left( {x_{A} ,t} \right)} & {{\text{q}} \ge \frac{1}{2}} \\ \end{array} } \\ \end{array} } \right.$$

where $f_{{\overline{\alpha }}} \left( {x_{A} ,{\text{t}} + \Delta {\text{t}}} \right)$ represents the post-collision and post-propagation state of the distribution function at time (${\text{t}} + \Delta {\text{t}}$) and the point $x_{A}$. ${\text{f}}_{{\upalpha }}^{{\text{c}}}$ is the value of the distribution function after a collision and before propagation state of the fluid node; the factor ${\text{q}}$ represents the normalized distance from the wall^31^. The factor ${\text{q}}$ is the normalized distance from the wall which equals to $\frac{{\left| {{\text{AC}}} \right|}}{{\left| {{\text{AB}}} \right|}}$ (Fig. 1, panel C, schematic diagram for one dimensional problem).

#### Wall shear stress

The total stress tensor for the fluid is as follows:

2 $$T_{ij} = - p.\delta_{ij} + \sigma_{ij}$$

where $p$, $\delta_{ij}$ and $\sigma_{ij}$ represent pressure, Kronecker symbol and contribution from the viscous force, respectively. $T_{ij} n_{j}$ represents the stress on the boundary surface element with normal vector $\vec{n}$. The wall stress vector, $\vec{\tau }$, is computed as:

3 $$\tau_{i} = T_{ij} n_{j} - (n_{j} T_{kj} n_{k} )n_{i}$$

The total stress ${\text{T}}_{{{\text{ij}}}}$ can be replaced by ${\upsigma }_{{{\text{ij}}}}$ since the projection of normal stress $\left( {{\text{p}}.{\updelta }_{{{\text{ij}}}} } \right)$ on the tangential plane is zero. The viscous stress for a Newtonian fluid is proportional to the strain rate tensor ($\sigma_{ij} = 2\mu \varepsilon_{ij}$)^47,48^ and is as follows:

4 $$\varepsilon_{ij} = - \frac{1}{{2\rho \tau c_{s}^{2} }}{\Pi }_{ij}$$

where ${\Pi }_{ij}$ represents a second order non equilibrium moment that can be computed locally from the particle distribution *functions*.

#### Model properties and boundary conditions

Flow conditions upstream and downstream of the local aortic region are heavily influential on local flow dynamics and must be accounted for. Additionally, the proper choice of boundary conditions is crucial as they are also influential on the accuracy of flow simulations. Blood was assumed to be a Newtonian and incompressible fluid with dynamic viscosity and density of 0.0035 Pa·s and 1050 kg/m^3^, respectively. The three patients with COA who received bypass grafting used our Doppler-based lumped parameter model that simulated the function of the left side of the heart to obtain the boundary conditions. We imposed the time-dependent inlet flow rate at the ascending aorta cross section and the outlet pressure at the descending aorta cross section using our lumped-parameter model (Fig. 1) in three patients with COA in both pre and post bypass grafting status. The velocity boundary condition, as introduced by Skordos ^49^and was implemented in OpenLB^50^ was assigned at the inlet. With this type of boundary conditions, velocity gradients of the boundary nodes are evaluated using a second-order finite difference scheme^51^. This information is then used to compute the off-equilibrium terms of the distribution functions^51^. Since the data locally available on corner and edge nodes are insufficient to evaluate the exact value of the pressure, the value of pressure on these nodes is extrapolated from bulk nodes^52^. Furthermore, in order to avoid pressure fluctuation artifacts at the inlet and make the simulation stable, the domain was initialized using a sinusoidal smooth start-up method^53,54^. In this method, the domain was solved assuming a half sinusoidal waveform for the inlet velocity. The domain state at the end of the half sinusoid was then used as the initial condition of the main problem with the true pulsatile flow waveform. The total flow rate going to the branches was calculated using the lumped-parameter model and was distributed to the branches based on their relative aortic cross-sectional areas at the inlet of each branch. A no-slip boundary condition was applied at the solid walls as described in the section above (*Modeling surface curvature near the wall of complex geometries*). Based on the knowledge that patients with COA are typically hypertensive and characterized by reduced compliance and elevated stiffness in both the proximal and distal aorta^55–57^, e.g., Jin et al.^58^, Keshavarz-Motamed et al.^26,29,59^ showed that a rigid-wall assumption for the aorta is reasonable and thus, the aortic wall in this study was treated as such.

The overall estimation of cardiac parameters is very dependent on the outputs of the lumped-parameter model due to the complex multiphysics nature of the aorta and heart valves. These lumped-parameter model outputs in-turn depend on the input parameters used in the lumped-parameter model. Our patient-specific, Doppler-based, lumped-parameter algorithm, which provided boundary conditions, has been validated against clinical catheterization data in patients with a substantial inter- and intra-patient variability with a wide range of disease^29,31,42^. We used the validated lumped-parameter model^29,31,42^ to obtain the boundary conditions in the present study.

#### Reconstructed geometries in patients with coarctation

The 3D geometries of the complete aorta (ascending aorta, aortic branches, descending aorta and bypass graft) were reconstructed from segmented CT images of patients using a 3-D image processing and model generation software package (Fig. 1), ITK-SNAP (version 3.8.0-BETA). Geometrical specifications of bypass grafts in patients 1, 2 and 3 were presented in Table 2. In order to parallelize the simulation, these 3-D reconstructions were voxelized into multiblocks which were distributed between computer processor units.

**Table 2:** Geometrical specifications of bypass grafts in patients 1, 2 and 3.

Patient No.	Bypass graft location	Bypass graft length (cm)	Bypass graft diameter
#1	Left subclavian artery to descending aorta	8.1	1.6
#2	Aortic arch to descending aorta	6.9	1.7
#3	Left subclavian artery to descending aorta	8.3	1.5

#### Numerical strategy

In order to stabilize complex turbulent fluid flow across the domain, single and multiple relaxation time LBM-based models were coupled with Smagorinsky’s turbulent model. We utilized second order accuracy method proposed by Bouzidi et al.^46^ to treat complex geometry. A smooth startup phase was added to the inlet velocity condition to suppress any undesired pressure fluctuation. Large Eddy Smagorinsky subgrid-scale model with constant $C_{s} = 0.1$ was applied^60^ for turbulent modelling. Mesh sensitivity analysis on spatial grid resolution was performed for maximum velocity and pressure drop at the coarctation region. Mesh definition was considered acceptable if no significant differences (lower than 2%) existed between successive mesh refinements in both quantities. An average of about 86 million cells were used depending on the size and morphology of the aorta. Maximum value of $y^{ + }$ was checked during the simulation to ensure that minimal lattice height is within the viscous sublayer. $y^{ + } = yu_{\tau } /v$ is the dimensionless height of the near-wall elements based on friction velocity ($u_{\tau } = \sqrt {\tau_{w} /\rho }$), where $u_{\tau } ,$ $y,$ $v$ and $\tau_{w}$ are friction velocity, normal cell distance away from the wall, kinematic viscosity and wall shear stress, respectively. For example, for patient #2, the calculated $y^{ + }$ was less than unity with the considered cell height of 100μm. The physical time step ($\Delta t$) was chosen based on the physical kinematic viscosity of blood $v_{phys}$ and the non-dimensional lattice kinematic viscosity $v_{{\text{lattice unit}}}$ as: $\Delta t = \frac{{v_{{\text{lattice unit}}} }}{{v_{{{\text{phys}}}} }}\Delta x^{2}$^61^. The physical time step was as low as 1.5 $\mu s$ in our simulations. In this study, we used Compute Canada, the Shared Hierarchical Academic Research Computing Network (SHARCNET: www.sharcnet.ca) that uses Intel Xeon (E5, E7, …) processors. The simulations were executed using the open-source library OpenLB, written in C +  + . Message Passing Interface (MPI) used for parallelization. For all simulations in this study, we typically used 50 nodes (32 to 40 cores per node) and the typical end to end simulation runtime was 1 h.

### Lumped parameter model

Our developed patient-specific Doppler-based lumped-parameter algorithm^29,42^ includes several sub-models, allowing analysis of complex coarctation disease in both pre and post bypass graft intervention and when coexistent with the other valvular, vascular and ventricular diseases. The input parameters for our Doppler-based lumped parameter algorithm can be reliably measured using two non-invasive techniques: Doppler echocardiography and the sphygmomanometer.

All details about modeling the left ventricle, left atrium, heart valves and computational algorithm were presented elsewhere^29,42^. Here, we provide concise description as follows:

#### Cardiac-arterial model

##### Left ventricle

LV pressure and volume were coupled using a measure of cardiac muscle stiffness, a time varying elastance E(t) calculated as follows:

5 $$E\left( t \right) = \frac{{P_{LV} \left( t \right)}}{{V\left( t \right) - V_{0} }}$$

where $P_{LV} \left( t \right)$, $V\left( t \right)$, and $V_{0}$ represent the LV time-varying pressure, time-varying volume, and unloaded volume, respectively.

As explained by Keshavarz-Motamed^42^, to represent the normalized elastance function of the LV, the double-Hill LV normalized time-varying elastance curves (E_N_) is calculated as follows^62,63^:

6 $$E_{N} \left( t \right) = N\left( {\frac{{\left( {\frac{t}{{\tau_{1} }}} \right)^{m1} }}{{1 + \left( {\frac{t}{{\tau_{1} }}} \right)^{m1} }}} \right)\left( {\frac{1}{{1 + \left( {\frac{t}{{\tau_{2} }}} \right)^{m2} }}} \right) + E_{min}$$

7 $$N = \frac{{E_{max} - E_{min} }}{2}$$

where $N$, $\tau_{1}$ , $\tau_{2}$, $m_{1}$, $m_{2}$, $E_{max}$ and $E_{min}$ represent the elastane normalization, ascending time translation, descending time translation, ascending gradient, descending gradient, maximum elastance and minimum elastance, respectively (see Table 1).

##### Left atrium

Following the same method described above for the LV model, LA pressure and volume were coupled using time varying elastance E(t), and thus the elastance function used for LA is defined in Eqs. 6 and 7 as well.

##### Modeling heart valves

***Aortic valve****.* The aortic valve was modeled using the analytical formulation for the net pressure gradient $\left( {PG_{net} } \right)$ across the aortic valve as follows:

8 $$\left. {PG_{net} } \right|_{AV} = \frac{2\pi \rho }{{\sqrt {\left. {E_{L} Co} \right|_{AV} } }}\frac{\partial Q\left( t \right)}{{\partial t}} + \frac{\rho }{{2\left. {E_{L} Co} \right|_{AV}^{2} }}Q^{2} \left( t \right)$$

and

9 $$\left. {E_{L} Co} \right|_{AV} = \frac{{\left( {\left. {EOA} \right|_{AV} } \right)A_{AO} }}{{A - \left. {EOA} \right|_{AV} }}$$

where ${\left.{E}_{L}Co\right|}_{AV}$,${\left.EOA\right|}_{AV}$,${A}_{AO}$,$\rho$ and $Q$ represent the valvular energy loss coefficient, the effective orifice area, ascending aorta cross sectional area, fluid density and transvalvular flow rate, respectively.

***Aortic regurgitation****.* Aortic regurgitation (AR) was modeled using the same analytical formulation as the aortic valve and is as follows:

10 $$\left. {PG_{net} } \right|_{AR} = \frac{2\pi \rho }{{\sqrt {\left. {E_{L} Co} \right|_{AR} } }}\frac{\partial Q\left( t \right)}{{\partial t}} + \frac{\rho }{{2\left. {E_{L} Co} \right|_{AR}^{2} }}Q^{2} \left( t \right)$$

and

11 $$\left. {E_{L} Co} \right|_{AR} = \frac{{EOA_{AR} A_{LVOT} }}{{A_{LVOT} - EOA_{AR} }}$$

where ${\left.{E}_{L}Co\right|}_{AR}$ , $EO{A}_{AR}$ and ${A}_{LVOT}$ represent regurgitation energy loss coefficient, regurgitant effective orifice area and LVOT area, respectively.

***Mitral valve***. The mitral valve (MV) was modeled using the net pressure gradient (${\left.P{G}_{net}\right|}_{MV}$) across the MV during LA ejection. ${\left.P{G}_{net}\right|}_{MV}$ was expressed as a function of $\rho$, ${Q}_{MV}$, $EO{A}_{MV}$ and ${M}_{MV}$, which represent the density of fluid, transvalvular flow rate, effective orifice area and inertance, respectively.

12 $$\left. {PG_{net} } \right|_{MR} = \frac{{M_{MV} }}{{EOA_{MV} }}\frac{{\partial Q_{MV} \left( t \right)}}{\partial t} + \frac{\rho }{{2\left. {EOA} \right|_{MV}^{2} }}Q_{MV}^{2} \left( t \right)$$

***Mitral regurgitation***. Mitral regurgitation (MR) was modeled using the following equation where the MR pressure gradient is calculated as the difference between mitral and LA pressure during systole.

13 $$\left. {PG_{net} } \right|_{MR} = \frac{{M_{MV} }}{{EOA_{MR} }}\frac{\partial Q\left( t \right)}{{\partial t}} + \frac{\rho }{{2\left. {EOA} \right|_{MR}^{2} }}Q^{2} \left( t \right)$$

where $\left. {EOA} \right|_{MR}$ represents the MR effective orifice area.

#### Modeling coarctation of the aorta

To model COA, two parallel branches were considered: (1) the first branch simulates the flow towards the upper body (aortic arch arteries); (2) a second branch simulates the flow crossing COA and directed towards the descending aorta. The second branch includes a resistance for the proximal descending aorta as well as a time-varying resistance and an inductance, together they represent the trans-coarctation net pressure gradient induced by the COA^29^:

14 $$\left. {TPG_{net} } \right|_{coa} = \frac{2\pi \rho }{{\sqrt {\left. {E_{L} Co} \right|_{coa} } }}\frac{\partial Q\left( t \right)}{{\partial t}} + \frac{\rho }{{2\left. {E_{L} Co} \right|_{coa}^{2} }}Q^{2} \left( t \right)$$

15 $$\left. {E_{L} Co} \right|_{COA} = \frac{{\left( {\left. {EOA} \right|_{coa} } \right)A}}{{A - \left. {EOA} \right|_{coa} }}$$

where ${\left.{E}_{L}Co\right|}_{coa}$ and ${\left.EOA\right|}_{coa}$ represent the energy loss coefficient of the COA and the effective orifice area of the COA, respectively. $A$, $\rho$, and $Q$ are the aortic cross-sectional area downstream of the COA, the fluid density and the trans-coarctation flow rate, respectively.

#### Modelling coarctation in presence of extra-anatomical bypass graft

When modelling COA for the patients who received bypass graft, it is important to quantify: (1) the flow towards the upper body (including aortic arch arteries); (2) the flow crossing the descending aorta. We modelled the flow in these patients by performing the following steps.

In the first step, we calculated the volume of blood passing through the descending aorta (flow passing COA + flow passing bypass graft) using Doppler echocardiography data ( $SV_{{\text{Descending aorta}}} = A_{{{\text{DAO}}}} \times VTI_{{{\text{DAO}}}}$; where $A_{DAO}$ and $VTI_{DAO}$ are the descending aorta area and the descending aorta velocity–time integral, respectively). Both $A_{{{\text{DAO}}}}$ and ${\text{VTI}}_{{{\text{DAO}}}}$ can be reliably measured using Doppler echocardiography. *Forward LVOT-SV,* defined as the total volume of blood that passes through the LVOT cross sectional area in every heartbeat, was calculated using Eq. 17 (see below). Therefore, flow through aortic arch branches was calculated by subtracting $SV_{{\text{Descending aorta}}}$ from *Forward LVOT-SV*.

In the second step, R_ub_ (upper body resistance, Table 1, Fig. 1) was adjusted so that the flow through aortic arch branches as well as descending aorta were equal to the ones calculated using Doppler echocardiography data.

#### Pulmonary flow

The pulmonary valve flow waveform was simulated by a rectified sine curve with duration ${t}_{ee}$ and amplitude Q_MPV_ as follows:

16 $$Q_{PV} \left( t \right) = Q_{MPV} \sin \left( {\frac{\pi t}{{t_{ee} }}} \right),t \le t_{ee} ;\;Q_{PV} \left( t \right) = 0\quad t_{ee} < t \leq T$$

where Q_MPV_, t_ee_ and T represent the mean flow rate of the pulmonary valve, end-ejection time and cardiac cycle duration, respectively. The only input flow condition in this study was Forward *LVOT-SV*. Indeed, in order for the lump-parameter algorithm to reproduce the measured Forward *LVOT*-SV, the mean flow rate of the pulmonary valve (Q_MPV_) was optimized.

#### Computational algorithm

The lumped-parameter model was analyzed numerically by creating and solving a system of ordinary differential equations in Matlab Simscape (MathWorks, Inc.), supplemented by additional functions written in Matlab and Simscape. To solve the system of differential equations, Matlab’s ode23t trapezoidal rule variable-step solver was used with an initial time step of 0.1 ms. The convergence residual criterion was set to 10^–6^ while initial voltages and currents of capacitors and inductors were set to zero. The model ran for ~ 150 cycles to reach a steady state before initiating the response optimization process as described below.

#### Input parameters

The developed algorithm uses the following input parameters measured using Doppler echocardiography: LV stroke volume, cardiac cycle duration, ascending aorta area, LVOT area, aortic valve effective orifice area, mitral valve effective orifice area, the effective orifice area of the COA, aortic cross-sectional area downstream of the COA, and grading of the severity of aortic and mitral valves regurgitation. Systolic and diastolic blood pressures, measured using a sphygmomanometer in the brachial artery of the arm, are additional input parameters for the developed algorithm.

#### Patient-specific response optimization

Four parameters of the model were optimized so that the lumped-parameter model reproduced the physiological measurements performed in the patient. The response optimization was performed in two sequential steps with tolerances of 10^–6^.

The mean flow rate of the pulmonary valve, Q_MPV_, could not be reliably measured using Doppler echocardiography. In the first step of optimization, Q_MPV_ was optimized to minimize the error between the *Forward LVOT-SV* calculated by the lumped-parameter model and the one measured in each patient since *Forward LVOT-SV* can be measured reliably using Doppler echocardiography.

In the second step of optimization, R_SA_, C_SAC_, and C_ao_ were optimized so that the maximum and minimum values of the aorta pressure were equal to the systolic and diastolic pressures, respectively, measured using a sphygmomanometer in the brachial artery of the arm in each patient. For the sake of simplicity, we considered the aortic resistance, ${R}_{ao}$, and the systemic vein resistance, ${R}_{SV}$, as constants and optimized the systemic arteries resistance, ${R}_{SA}$, as the main contributor of the total systemic resistance. This is reasonable since the left ventricle faces the total systemic resistance rather than the individual resistances, and the systemic arteries resistance is one order of magnitude greater than both the aortic resistance and systemic vein resistance.

In addition, we conducted an extensive parameter sensitivity analysis that concluded the pulmonary parameters (e.g., C_PVC_) have negligible effects on the model output variables. Therefore, we did not include these pulmonary parameters in the parameter-optimization process and considered them as constants given in Table 1.

### Four-dimensional flow magnetic resonance imaging (4-D flow MRI)

Four-dimensional flow magnetic resonance imaging (4-D flow MRI) is a recent development of phase-contrast MRI (PC-MRI) with the capability to comprehensively assess blood flow in three spatial dimensions over the cardiac cycle^64^. 4-D flow MRI provides visualization of the vascular territory of interest and allows for the estimation of hemodynamic biomarkers such as wall shear forces^65^ and pressure gradients^66,67^. Furthermore, 4-D flow MRI provides comprehensive information regarding complex flow patterns in vascular diseases^68^*.* In this study, acquisition of 4-D flow MRI data in five patients with COA was performed (Fig. 1, Panel B, data acquisition and analysis workflow of 4-D flow MRI) by standard Cartesian 4-D flow sequence using 1.5 T MRI scanners (Philips Achieva; Philips Medical Systems, Best, the Netherlands). Electrocardiogram gating synchronized and diaphragm navigator gated 4-D flow MRI were performed during free breathing. Acquisition parameters were as follows: spatial resolution of (1.97–2.62, 1.97–2.62, 2.5–4 mm^3^), temporal resolution of 36–40 ms. Velocity encoding was set to the range (1.5–4.5 m/s), the total scan time for each measurement varied from 8 to 15 min. All 4-D flow data was corrected for multiple sources of phase offset errors and noises such as velocity aliasing, Maxwell terms, and eddy currents using an in-house MATLAB-based code (MathWorks, Inc.). Velocity field extracted from 4D flow MRI measurements was smoothed using a multidimensional spline smoothing method suggested by Garcia^69,70^. The algorithm deals with occurrences of missing and outlying values and removes random errors automatically that allows rapid smoothing of the data and can replace spurious or missing vectors with the smoothed ones. The algorithm could deal with a large amount of missing data and reduce the experimental noise while keeping the most important features of a dataset^69^. In the current study, to investigate the effect of resolution and to compare LBM and 4D flow MRI velocity fields on identical grids, we down sampled the high-resolution LBM velocity fields by linear interpolation on the 4D flow MRI grid (Fig. 1, panel B, Schematic diagram).

Using ITK-SNAP (Yushkevich et al., 2006; http://www.itksnap.org) and an in-house MATLAB-based code, the 3-D segmentation of the thoracic aorta geometry and orifice shape of aortic stenosis or bicuspid valve was performed. In order to smooth the geometry and fix any defect^71^, Fusion 360 (Autodesk, Inc) and Meshmixer (Autodesk, Inc) were used. Finally, the Stereolithography (STL) format of the geometry (domain) was extracted for the us in our computational simulation. Due to the lack of echocardiography data, the Doppler-based lumped parameter algorithm did not provide us the boundary conditions. We did not use the 4-D flow MRI velocity field as the initial condition of the domain. We only used time-varying flow velocity information at the ascending aorta inlet and three branches, extracted from 4-D flow MRI data to define the boundary condition in addition to the pressure outlet boundary condition at the descending aorta outlet. Only the velocity field calculations of the *Lattice Boltzmann model* were validated against 4-D flow MRI measurements in five COA patients. We down sampled the high-resolution LBM velocities into PC-MRI resolution by linear interpolation of LBM velocity on MRI sub grid in order to study the effect of resolution and comparing LBM to 4-D flow MRI velocity fields on identical grids. Moreover, the down-sampled LBM data was subjected to an imitation of the smoothing inherent in the 4-D flow MRI measurement in order to have the closest LBM approximation to the 4-D flow MRI data. The down-sampling and smoothing procedures are schematically shown in Fig. 1 (Panel B).

Deidentified and anonymous patients at Stephenson Cardiac Imaging Centre, Libin Cardiovascular Institute of Alberta (Calgary, AB, Canada) were considered. Informed consent was obtained from all patients. The protocol was reviewed and approved by the Ethics Committee of the institution (Libin Cardiovascular Committee). The selections were done by operators blinded to the objectives and contents of this study at each institution and the protocols were reviewed and approved by the Institutional Review Boards of each institution. All methods and measurements were performed in accordance with relevant guidelines and regulations including guidelines of the American College of Cardiology and American Heart Association.

## Results

### Validation

The velocity fields calculated using Lattice Boltzmann model were validated against 4-D flow MRI measurements in five patients with COA. The level of qualitative agreement of the velocity field between 4D flow MRI data and LBM results, that we observed in this study, is comparable to those of previous observations made using other computational fluid dynamics methods^72–75^. Bland–Altman plots were graphed to describe the degree of concordance and agreement between LBM and 4-D flow MRI measurements^76^. Figure 2 (Panel A) describes sample cases of voxel-by-voxel Bland–Altman analysis between the velocity fields resulted from the smooth down sampled low-resolution LBM and 4-D flow MRI measurements on the entire flow domain at the peak systole. The simulated velocity fields were in agreement with the velocity fields measured using 4-D flow MRI in patients: as examples, biases (means of differences) were − 0.05, − 0.04, − 0.0087, − 0.046 and 0.057 [m/s] and corresponding limits of agreement (1.96 SD) were also ± 0.192, ± 0.28, ± 0.46, ± 0.35 and ± 0.374 [m/s] for patients #1, #2, #3, #4 and #5, respectively.

Moreover, Fig. 2 (Panel B) shows the statistical analyses of the planar velocity differences between velocity field resulted from our computational framework and 4-D flow MRI measurements in sample cases at the peak of systole. As this figure shows, the 4-D flow MRI velocity field and the down sampled LBM-based velocity fields were compared using Pearson’s correlation and Bland–Altman analysis. The coefficient of determination (R^2^) was used to assess the linearity between the results from 4-D flow MRI and our computational framework at these planar sections. The coefficients of determination were 0.9508, 0.9305, 0.9126, 0.574 and 0.592 for patients #1, #2, #3, #4 and #5, respectively. Lateral section Biases (means of differences) were also − 0.089, − 0.032, 0.082, − 0.028 and − 0.07 m/s and the corresponding limits of agreement (1.96 SD) were ± 0.066, ± 0.186, ± 0.171, ± 0.369 and ± 0.294 m/s for patients #1, #2, #3, #4 and #5, respectively, which shows agreements between the data resulted from 4-D flow MRI and our computational framework. All the statistical correlations between the LBM results and 4D Flow MRI measurements are in the range of previous observations^73–75^. Although 4-D flow MRI enables measurement of blood flow velocity in vivo, it has some limitations that can be responsible for the differences of its results with the LBM results. Some important limitations of 4-D flow MRI are the following: (1) low temporal resolution (20 ms highest)^77^; (2) natural movements of the heart and lungs causes motion artifacts in the measurements data (2) inaccurate velocity measurements at the boundary of stationary and moving tissues due to noisy phase shifts; and (3) the low spatial resolution of 4-D flow MRI does not allow capturing the turbulent characteristics at the sub-voxel scale^64,75,78^. The largest discrepancies between the velocity fields obtained using LBM and 4-D flow MRI were found at locations where MRI could only provide velocities with fluctuation artefacts. Intravoxel dephasing and missing turbulent characteristics at the sub-voxel scale have been concluded to be responsible for these fluctuation artefacts^64,75,78^.

In addition, our Doppler-based lumped-parameter model calculations were validated against clinical cardiac catheterization data (the instantaneous pressures in the aorta and LV) in patients with complex valvular, ventricular and vascular diseases with a substantial inter- and intra-patient variability with a wide range of disease (*N* = 49)^42,77^. The lumped-parameter model has already been validated against in vivo cardiac catheterization in patients with coarctation (*N* = 40)^29,31^ and some sub-models have been validated against in vivo MRI data (*N* = 57)^79^. In addition, some of the sub-models of the lumped parameter model have been used previously^27,42,59,79–86^. Moreover, the entire computational framework (Lattice Boltzmann method and lumped parameter model) was validated against clinical Doppler echocardiography in this study for the three patients with COA in pre- and post bypass graft status. We observed good agreements between the simulated and clinical peak Doppler echocardiography pressure gradients with the mean relative error of 3.9% (Δ*P* = 4V_max_^2^; V_max_: maximum velocity downstream of COA during systole).

### Velocity

**Figure 3:**
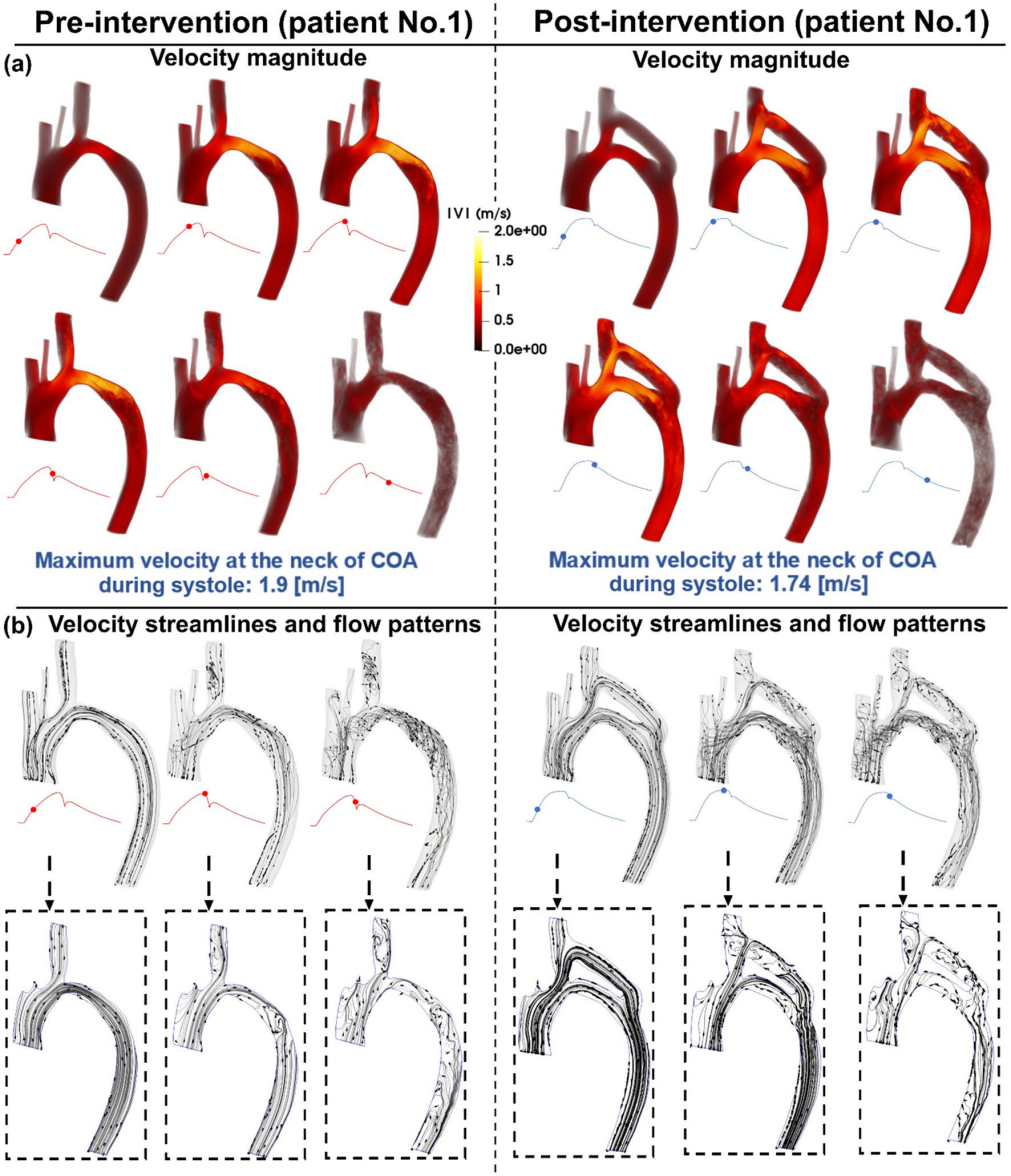
Flow modeling in Patient No. 1 in pre and post intervention status. (**a**) Time-evolving velocity magnitude at six instances; (**b**) 3-D (upper row) and 2-D (lower row) streamlines through the aorta at three instances. *Baseline characteristics (Patient No. 1):* mild-moderate aortic stenosis, mild aortic valve regurgitation, trace mitral regurgitation, COA and hypertension.

Figures 3, 5, and 7 (panels A) show the velocity mappings of the aorta in both pre- and post-intervention status for patients #1, #2, and #3, respectively. Patient #1 (Fig. 3, panel A) exhibits elevated velocity strictly at the neck of COA, reaching a maximum of 1.9 [m/s] pre-intervention which drops slightly to 1.74 [m/s] post-intervention. Furthermore, increased velocity magnitudes are observed at the inlet of the graft as well as at the neck of COA post-intervention. The velocity through the graft appears to reach a maximum of approximately 1.4 [m/s]. Overall, the bypass graft slightly reduced the maximum observed velocity in patient #1, however, there is no substantial improvement. In contrast to the isolated regions of elevated velocity in patient #1, patient #2 (Fig. 5, panel A) exhibits an elevated velocity at the sight of COA, reaching a maximum of 1.59 [m/s] pre-intervention. Post-intervention, the condition is critically improved as the maximum recorded velocity is 1.03 [m/s] with a maximum of 0.6 [m/s] through the graft. Elevated velocities post-intervention are mainly visualized at the inlet and outlet of the graft as well as distal to the outlet. Patient #3 (Fig. 7, panel A) exhibits elevated velocities in nearly all regions of the aorta pre-intervention, with a maximum velocity of 1.47 [m/s] recorded at the neck of COA. The condition is substantially improved as the maximum velocity post-intervention decreases to 1.05 [m/s] with a maximum of 0.7 [m/s] through the graft, however, elevated velocities are observed in most regions of the aorta, similar to the pre-intervention status.

### Streamlines and evolution of flow patterns

Figures 3, 5, and 7, panels B illustrate the velocity streamlines and time evolving flow patterns in the central plane pre-intervention for patients #1, #2, and #3, respectively. All patients exhibited relatively laminar flow patterns throughout the early systole. In all three examined patients, chaotic flow ensues throughout peak systole. A small vortex appears directly downstream of the COA in patient #1 (Fig. 3, panel B), which later dissipates during late systole. Similarly, a strong vortex appears downstream of the COA in patient #3 (Fig. 7, panel B) which also dissipates in the late systole. Both vortices lead to recirculation and reversed flow—the flow patterns are disturbed in the descending aorta in late systole as well (patient #2, Fig. 5, panel B).

Post-intervention, in all three patients, the flow is laminar through all regions of the graft and the aorta during early systole. The flow patterns in all three patients exhibited different behaviors in the remaining systolic phase. The flow patterns in patient #1 (Fig. 3, Panel B) become more chaotic through the graft and at the outlet during peak systole leading to some recirculation and reversed flow. In the late systole, the flow pattern throughout the entire aorta and bypass graft is complicated with a large amount of reversed flow and recirculation. Patient #2 (Fig. 5, panel B) exhibits relatively laminar flow patterns during early systole with increased disturbed flow in late systole. In patient #3 (Fig. 7, panel B), a large vortex formed at the inlet of the graft and an additional vortex was observed downstream of the COA. Flow remains chaotic in the late systole upstream of the COA, the vortex at the inlet of the graft was dissipated and laminar flow was mostly restored throughout the graft. However, the vortex downstream of the COA expanded circumferentially and collected all flow from the graft.

Overall, the bypass graft in patient #1 does not seem to improve flow patterns except for resolving the vortex in peak systole, the behavior of the velocity streamlines is similar in each phase of pre-intervention compared to post-intervention status. Intervention in patient #2 did not seem to have a significant effect on the velocity streamlines and flow patterns, however, it did not worsen the condition for this patient who already exhibited laminar flow patterns. In patient #3, although the graft appears to slightly improve the flow patterns in the early systole by evenly distributing the flow across the aorta, the flow becomes more chaotic with larger vortices in the other phases of systole compared to the pre-intervention status.

### Viscous shear stress

**Figure 4:**
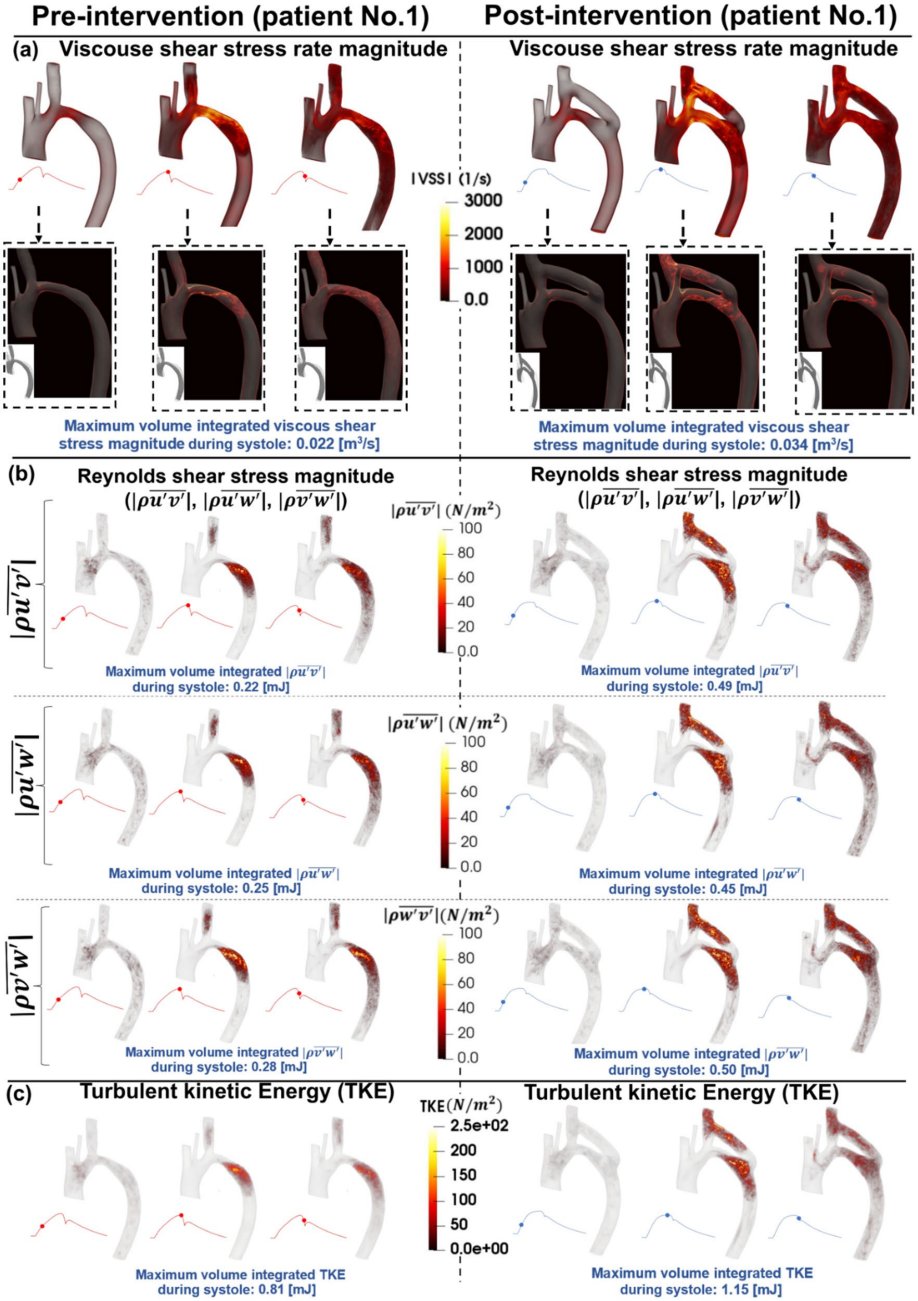
Flow modeling in Patient No. 1 in pre and post intervention status. (**a**) Viscous shear stress (VSS) magnitude; (**b**) Computed Reynolds Shear stress ($\rho \overline{{u^{\prime}v^{\prime}}} , \rho \overline{{u^{\prime}w^{\prime}}}$ and $\rho \overline{{w^{\prime}v^{\prime}}}$) magnitude; (**c**) Turbulent kinetic energy (TKE), computed as $\frac{1}{2}\rho \left( {\overline{{u^{{\prime}{2}} }} + \overline{{v^{{\prime}{2}} }} + \overline{{w^{{\prime}{2}} }} } \right)$, where u, v, w and ρ correspond to the three components of the instantaneous velocity vector and density. The bar and prime denote the ensemble averaged and fluctuating components, respectively.

The streamlines and velocity profile presented above clearly demonstrate the main features of the flow, but do not highlight the intensity of the spatial velocity gradients. Viscous shear stress (VSS) quantifies the effect of shearing between adjacent layers of fluid. In Figs. 4, 6, and 8 (panels A), VSS contours are shown in pre-intervention status for patients #1, #2, and #3, respectively. Slightly elevated VSS is observed directly at the neck of COA with negligible VSS values in all other regions for all three patients throughout the early systole. During peak systole, VSS rapidly increased upstream, at the neck, and downstream of the COA, reaching a maximum magnitude of 0.022 [m^3^/s], 0.018 [m^3^/s], and 0.030 [m^3^/s] pre-intervention in patients 1–3, respectively. VSS slightly dissipated throughout the late systole, however, elevated levels did remain through all regions of the examined aorta.

**Figure 5:**
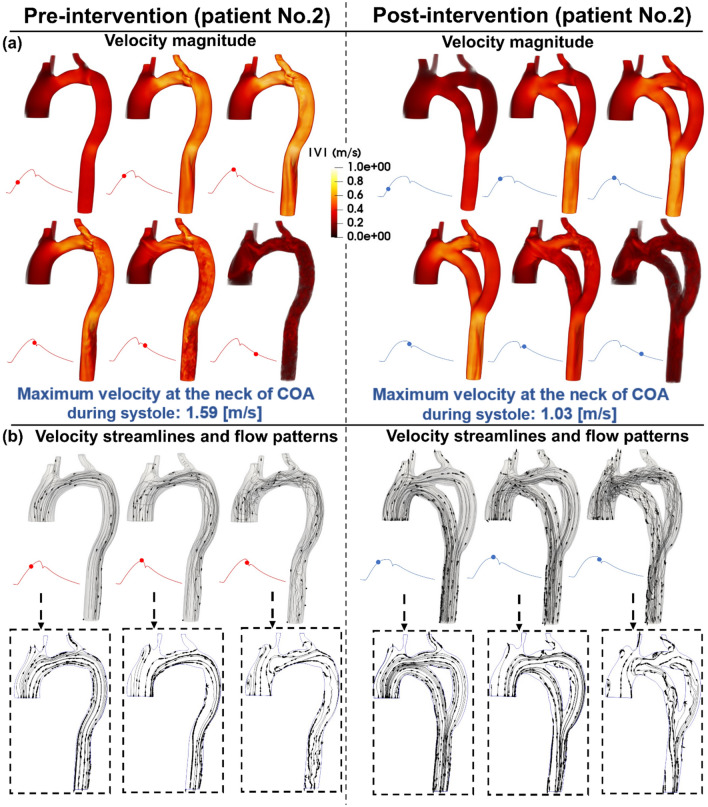
Flow modeling in Patient No. 2 in pre and post intervention status. (**a**) Time-evolving velocity magnitude at six instances; (**b**) 3-D (upper row) and 2-D (lower row) streamlines through the aorta at three instances. *Baseline characteristics (Patient No. 2):* COA and hypertension.

**Figure 6:**
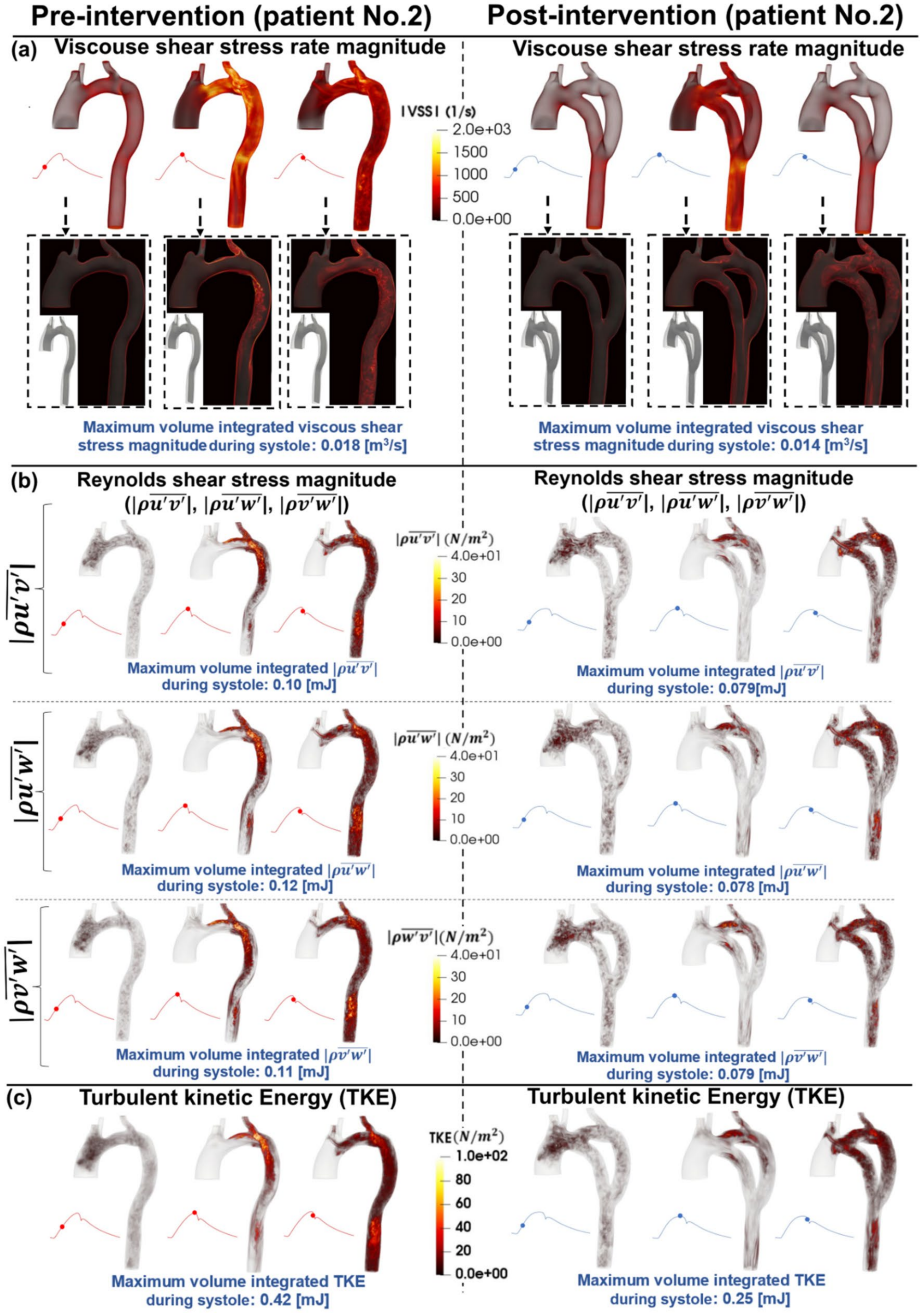
Flow modeling in Patient No. 2 in pre and post intervention status. (**a**) Viscous shear stress (VSS) magnitude; (**b**) Computed Reynolds Shear stress ($\rho \overline{{u^{\prime}v^{\prime}}} , \rho \overline{{u^{\prime}w^{\prime}}}$ and $\rho \overline{{w^{\prime}v^{\prime}}}$) magnitude; (**c**) Turbulent kinetic energy (TKE), computed as $\frac{1}{2}\rho \left( {\overline{{u^{{\prime}{2}} }} + \overline{{v^{{\prime}{2}} }} + \overline{{w^{{\prime}{2}} }} } \right)$, where u, v, w and ρ correspond to the three components of the instantaneous velocity vector and density. The bar and prime denote the ensemble averaged and fluctuating components, respectively.

**Figure 7:**
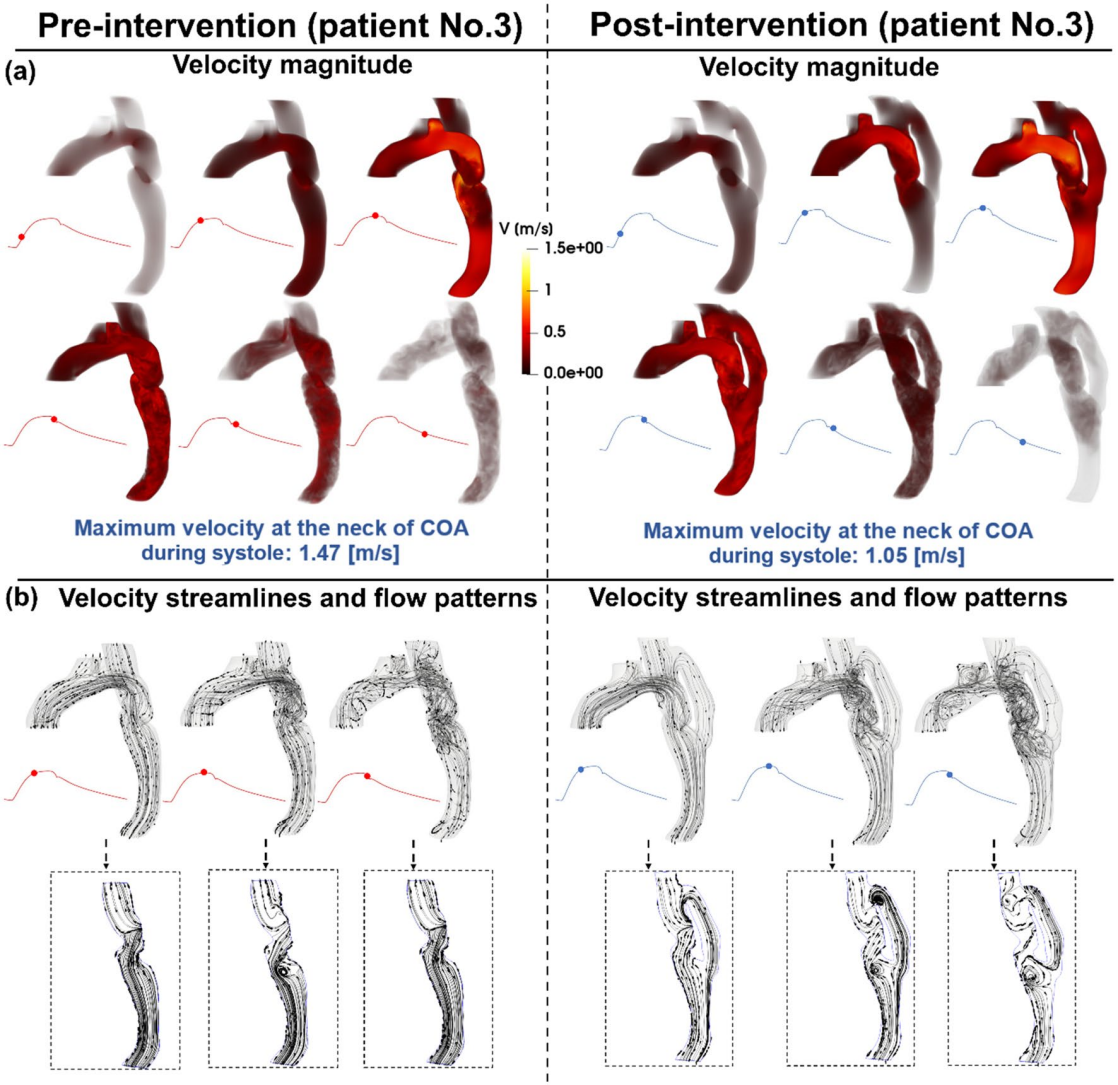
Flow modeling in Patient No. 3 in pre and post intervention status. (**a**) Time-evolving velocity magnitude at six instances; (**b**) 3-D (upper row) and 2-D (lower row) streamlines through the aorta at three instances. *Baseline characteristics (Patient No. 1):* moderate aortic stenosis, mild mitral regurgitation, severe COA, descending aorta aneurysm, and hypertension.

**Figure 8:**
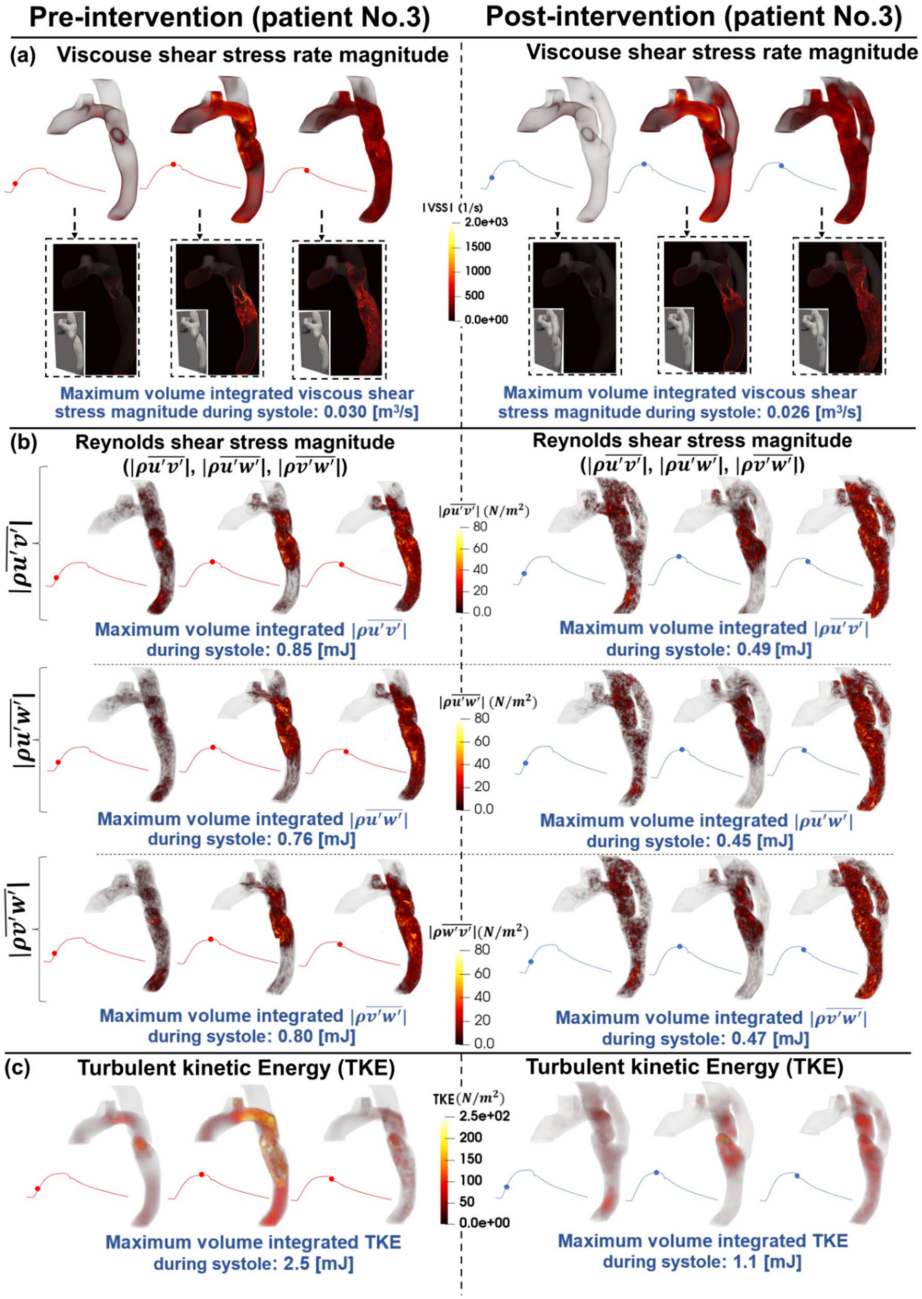
Flow modeling in Patient No. 3 in pre and post intervention status. (**a**) Viscous shear stress (VSS) magnitude; (**b**) Computed Reynolds Shear stress ($\rho \overline{{u^{\prime}v^{\prime}}} , \rho \overline{{u^{\prime}w^{\prime}}}$ and $\rho \overline{{w^{\prime}v^{\prime}}}$) magnitude; (**c**) Turbulent kinetic energy (TKE), computed as $\frac{1}{2}\rho \left( {\overline{{u^{{\prime}{2}} }} + \overline{{v^{{\prime}{2}} }} + \overline{{w^{{\prime}{2}} }} } \right)$, where u, v, w and ρ correspond to the three components of the instantaneous velocity vector and density. The bar and prime denote the ensemble averaged and fluctuating components, respectively.

Post-intervention, during early systole, all 3 patients had a similar behavior as the pre-intervention state, with slightly elevated VSS around the neck of COA. In patient #1 (Fig. 4, panel A), substantially elevated VSS was observed at the inlet of the graft and through the neck of COA reaching a maximum of 0.034 [m^3^/s] at peak systole, which was much greater than pre-intervention (0.022 [m^3^/s] at peak systole). Patient #2 (Fig. 6, panel A) displayed slightly elevated VSS in all regions of the aorta at peak systole compared to early systole, with a more substantial increase at the outlet of the graft. Maximum VSS recorded for patient #2 was 0.014 [m^3^/s] which was slightly improved when compared to the pre-intervention status (0.018 [m^3^/s]). Patient #3 (Fig. 7, panel A) exhibited similar VSS patterns to pre-intervention, with increased VSS upstream, at the neck, and downstream of the COA reaching a maximum of 0.026 [m^3^/s] in post-intervention status at peak systole which was slightly improved from pre-intervention (0.030 [m^3^/s]). All three patients exhibited a similar behavior with regards to VSS in the late systole as the patterns remained similar to peak systole with smaller magnitudes.

### Turbulent characteristics

While blood flow is generally laminar in healthy vessels, it may experience transition to turbulence under pathophysiological conditions. In this study, turbulent kinetic energy (TKE) and Reynolds shear stress (RSS), which are both derived using fluctuating components of the flow velocities, were investigated to characterize the level of fluctuations in the flow field through the aorta as follows:

#### Reynolds shear stress

In Figs. 4, 6, and 8 (panels B), the RSS mapping is shown the pre-intervention status for patients #1, #2, and #3, respectively. Consistently low or negligible RSS was observed in the early systole for all 3 patients. In peak systole, patients #1 (Fig. 4, panel B) and #2 (Fig. 6, panel B) exhibited RSS isolated to the neck of COA and slightly downstream whereas patient #3 (Fig. 8, panel B) exhibited increased RSS upstream from the COA as well. Throughout late systole, the RSS remained in the same regions as during peak systole for patient #1 with a decrease in magnitude, however, for patients #2 and #3, the regions of RSS expanded down the descending aorta. Maximum RSS values observed in patients #1, #2, and #3 were 0.22–0.28 [mJ], 0.10–0.12 [mJ], and 0.76–0.85 [mJ], respectively.

Post-intervention, similar to pre-intervention, RSS levels during the early systole were low for all 3 patients. Patient #1 (Fig. 4, panel B) displays increased RSS (significant worsening) at peak systole compared to pre-intervention (with a maximum range of 0.22–0.28 [mJ]) with elevated levels at the neck of COA along with the inlet and outlet of the graft achieving a maximum range of 0.45–0.50 [mJ] in post-intervention status. The regions of elevated RSS remained constant through the late systole with decreased magnitudes. Unlike patient #1, patient #2 (Fig. 6, panel B) displayed reduced RSS at peak systole with a maximum range of 0.078–0.079 [mJ] in post-intervention status, compared to pre-intervention (with a maximum range of 0.10–0.12 [mJ]). Patient #3 (Fig. 8, panel B) had a significant reduction (improvement) in RSS post-intervention (maximum RSS observed ranges: pre-intervention: 0.76 to 0.85 [mJ]; post-intervention: 0.45 to 0.49 [mJ]).

Overall, the condition for patient #1 worsened post-intervention as there were more observed regions of elevated RSS. In contrast, the condition for patient #2 greatly improved post-intervention, not only the maximum magnitudes of RSS decrease, but the regions of elevated RSS did not cover as much area. The RSS patterns for patient #3 and contours remained very similar post-intervention, however, the magnitudes were greatly decreased resulting in improved RSS.

#### Turbulent kinetic energy

Figures 4, 6, and 8 (panels C) illustrate the TKE contours throughout the aorta pre-intervention for patients #1, #2, and #3, respectively. In the early systole, for all three patients, there were negligible amounts of TKE throughout the aorta. In patients #1 (Fig. 4, panel C) and #2 (Fig. 6, panel C), the TKE observed at peak systole was isolated to the neck of COA and directly downstream, reaching a maximum of 0.81 [mJ] and 0.42 [mJ], respectively. The elevated levels of TKE dissipate in patient #1. However, in patient #2, the TKE appeared to expand to most parts of the aorta with slightly decreased magnitude from peak systole. In contrast to the patients #1 and #2, at the peak systole, the TKE contours observed in patient #3 (Fig. 8, panel C) were observed upstream, at the neck, and downstream of the COA reaching a maximum of 2.5 [mJ]. In the late systole, nearly all TKE dissipates for all three patients.

Post-intervention, similar to pre-intervention, all three patients displayed very low TKE in the early systole. The behavior of TKE for the 3 patients varied significantly throughout peak systole and late systole. Patient #1 (Fig. 4, panel C) exhibited elevated TKE at the neck of COA as well as at the inlet and outlets of the graft reaching a maximum of 1.15 [mJ] in post-intervention compared to pre-intervention (0.81 [mJ]). Throughout late systole, TKE remained present in the same regions with decreased magnitudes. In patient #2 (Fig. 6, panel C), TKE was low throughout peak systole with slightly elevated levels at the proximal section of the bypassed aorta. TKE slightly increases throughout most parts of the aorta and graft in the late systole. However, magnitudes in post-intervention status (with a maximum of 0.25 [mJ]) were much lower than in pre-intervention status (with a maximum of 0.42 [mJ]). TKE contours shown for patient #3 (Fig. 8, panel C) exhibited slightly elevated levels directly around and at the neck of COA during peak systole, with negligible levels in the graft. In the late systole, elevated TKE expanded throughout more of the graft, while remaining at a low magnitude compared to pre-intervention. The maximum recorded TKEs were 2.5 [mJ] and 1.1 [mJ] in pre and post-intervention status, respectively, in patient #3 throughout systole.

It is evident that the TKE values are significantly higher post-intervention for patient #1 which may have negative implications in the future. It is important to note that high levels of TKE were recorded at both the inlet and outlet of the graft. The condition for patient #2 improved with a great reduction in TKE, and minimal levels through the graft. Furthermore, for patient #3, the implemented bypass graft greatly improved the TKE expressed in the regions around COA—Maximum values greatly decreased, and the flow was much less turbulent compared to pre-intervention with negligible turbulence detected through the graft.

## Discussions

Accurate hemodynamic analysis is not only important for the diagnosis of COA, but treatment decisions also rely heavily on the hemodynamics assessment in both pre and post intervention states to minimize patient risks. Our results may provide insights about possible reasons for graft failure as follows:

### Bypass grafting may not improve wall shear stress

**Figure 9:**
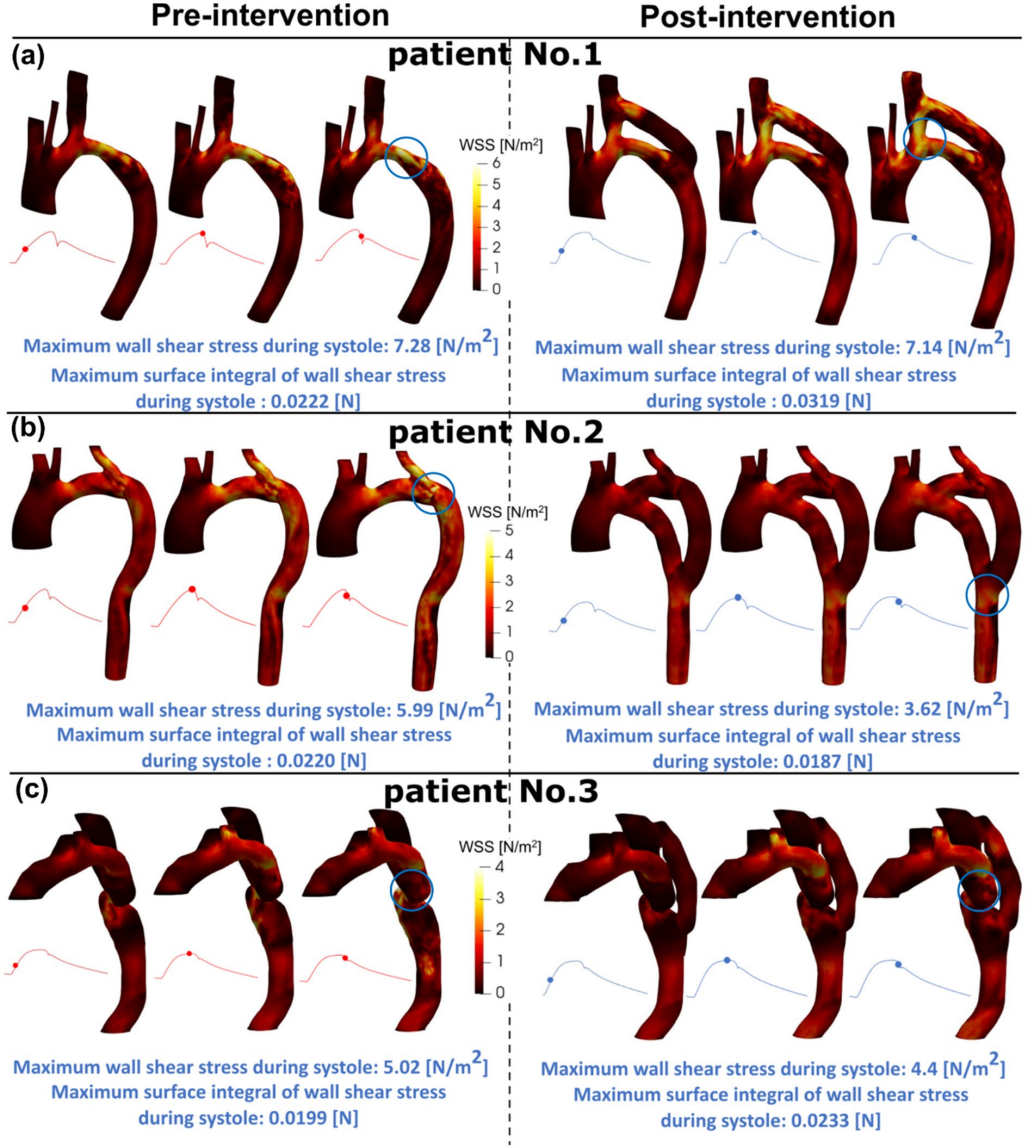
Flow modeling in Patients No. 1, 2 and 3 in pre and post intervention status. (**a**) Wall shear stress in patient #1; (**b**) Wall shear stress in patient #2; (**c**) Wall shear stress in patient #3. Blue circles indicate maximum wall shear stress locations.

The presence of the COA itself largely alter the flow dynamics which in turn contributed to elevated wall shear stress mainly at the COA region as well as distal to the COA. Wall shear stress, as a force induced by blood flow, has a major impact on regulating endothelial function and is a predeterminant biomarker of disease progression. High abnormal stresses (e.g., elevated wall shear stress) can result in endothelial dysfunction, dedifferentiation of arterial smooth muscle and medial thickening^26,28,80,87–94^. We observed that bypass graft exacerbated aortic wall shear stress near the junctions of anastomoses for all 3 patients investigated in this study (Fig. 9). In addition, bypass grafting does not always alleviate high wall shear stress significantly. As one example, in patient #1, maximum wall shear stress during systole did not improve post intervention (pre-intervention: 7.28 N/m^2^; post-intervention: 7.14 N/m^2^) and maximum surface integral of wall shear stress during systole increased significantly post intervention (pre-intervention: 0.0222 N/m^2^; post-intervention: 0.0319 N/m^2^). Such exposures of endothelial cells to high shear stress may affect vessel distensibility and compliance and potentially may lead to arterial remodeling, aneurysm, rupture and dissection.

### Patients with COA have higher incidence of hypertension after bypass graft intervention

Patients with COA usually have upper extremity hypertension and are characterized by reduced systemic arterial compliance ^29,55,57,95^. The post-intervention data collected in patients shows that the brachial pressure increased (Table 3). Consistently, we observed that the systemic arterial compliance was further reduced in patients #1 and #3 while it was not improved in patient #2 after bypass grafting (Table 3). Our findings suggest that although introducing bypass graft may reduce the flow resistance, it may also reduce the systemic compliance leading to hypertension.

**Table 3:** Hemodynamics parameters in 3 COA patients who underwent bypass grafting.

Patient No.	Status	Systolic brachial pressure (mmHg)	Diastolic brachial pressure (mmHg)	Peak Doppler pressure gradient (mmHg)	Systemic arterial compliance (mL/mmHg)	Total stroke volume (mL)	Flow rate through branches (mL)	Flow rate through contraction (mL)	Flow rate through bypass graft (mL)
#1	Pre intervention	131	62	14.4	1.24	85	35.70	49.30	N/A
	Post intervention	145	62	12.1	1.00	83	24.07	42.00	16.90
#2	Pre intervention	150	67	10.11	0.73	61	20.74	40.26	N/A
	Post intervention	143	53	4.1	0.73	66.5	14.63	27.60	24.00
#3	Pre intervention	144	78	8.64	1.30	86	30.80	55.20	N/A
	Post intervention	148	71	4.2	1.07	82.5	22.28	47.70	12.53

Note 1: Peak Doppler pressure gradient (Δ*P* = 4 V_max_^2^; V_max_: maximum velocity downstream of COA during systole). Note 2: Systemic arterial compliance (SAC = SV/PP; SV: total stroke volume, PP: pulse pressure (diastolic brachial pressure–systolic brachial pressure)).

### Bypass graft may lead to intimal hyperplasia due to the persistent abnormal aortic hemodynamics

Intimal hyperplasia is a common cause for graft failure which involves the abnormal layering of cells on a blood vessel membrane across a vascular reconstruction (e.g., a bypass graft)^18,19,96^. This phenomenon is supposed to be an adaptation process following an intervention; however, the cell proliferation and differentiation process causes the region of connection between the vessel and graft to narrow, *causing recurrent COA*, typically near the junctions of anastomoses^18,19,96,97^. Moreover, graft implementation may result in local turbulence which may contribute to the progression of intimal hyperplasia near the junctions of anastomoses, specifically at the outlet^18,97–99^. In response to abnormal fluid dynamics, the vessel wall attempts to correct the flow disruption by intimal thickening, causing the narrowing of the vessel^18,97–99^. It has also been shown that abnormal flow velocity has been linked with high cell turnover, leaky cell junctions, platelet aggregation and smooth muscle proliferation, all of which contribute to intimal hyperplasia^38^. Once this process begins post-intervention, it is difficult to control, leading to a significant risk for any vascular reconstructive surgery^96^.

Our findings suggest that bypass grafting may improve hemodynamic metrics (reduction in shear stresses, flow velocities, and turbulent characteristics)^18,38,97–99^ in some patients. However, the hemodynamic conditions worsened in others which has previously been linked to the onset and progression of intimal hyperplasia despite reduction in Doppler pressure gradient (Table 3). In addition, the geometry of the bypass graft may significantly impact blood flow parameters such as the flow field and wall shear stress (Fig. 9) which often lead to the development and progression of intimal hyperplasia near the junctions of anastomoses^37–39,97^. Based on the results of this study, it seems that patient #1 may be at risk of intimal hyperplasia post-intervention. The flow velocity magnitude did not significantly improve in patient #1 post-intervention and indeed, elevated flow velocity may lead to arterial wall complications. Moreover, bypass graft did not reduce the flow rate substantially through the COA (pre: 49 mL, post: 42 mL; Table 3). Furthermore, viscous shear stress as well as turbulence characteristics (such as Reynold’s shear and turbulent kinetic energy) increased post-intervention significantly which may lead to an increased risk of graft failure in patient #1. The hemodynamic conditions improved for patients #2 and #3 for most of the analyzed hemodynamic metrics. Despite this, both patients may be at risk for developing intimal hyperplasia due to the elevated abnormal hemodynamics near the junctions of anastomoses.

### Bypass graft may lead to pseudoaneurysm formation and potential aortic rupture due to aortic wall disruptions

A pseudoaneurysm, or false aneurysm, is a common long-term complication following a bypass graft surgery for COA and occurs in 11–24% of cases^21,24,100,101^. Although infrequent early after intervention, pseudoaneurysm incidence increases over-time and are commonly diagnosed during long-term follow-up^24^ occurring near the junctions of anastomoses^24,102–104^. Aortic pseudoaneurysms are often the result of a disruption of at least one layer of the aortic wall, which is contained within the remaining vascular layers and surrounding structures^103^. Disturbed flow conditions, including elevated velocity flow, high wall shear stresses and vortical flow^105^, may lead to wall complications and pseudoaneurysm^103,106^. If left untreated, pseudoaneurysms are likely to result in mortality or loss of a limb^24,107^. Indeed, monitoring of fluid dynamics at the inlet and outlet of bypass grafts is crucial to prevent potential pseudoaneurysm rupture. Our findings in this study suggest that all patients investigated in this study exhibit at least some of the following abnormal hemodynamics parameters: elevated velocity magnitude, persistence of vortical flow structure, elevated turbulence characteristics, and elevated wall shear stress (Figs. 3, 4, 5, 6, 7, 8, 9) at the bypass graft junctions^105^. All the mentioned abnormal hemodynamic metrics may lead to the formation and potential rupture of pseudoaneurysm.

### Use of wall shear stress for diagnosis and intervention planning

The correlations of velocity and wall shear stress (WSS) with vascular disease progression have been recognized since 1995^108^. Although WSS is not yet established in clinical practice as a marker of intervention success, several recent clinical studies raised attention to it specifically towards the correlation of WSS with ascending aorta dilation and rupture^109–114^. All these studies^109–114^ addressed (in large population data) the importance of WSS assessment in order to predict disease progression, such as for the prediction of aortic dilation rate. According to one recent clinical study^114^, WSS was assessed in for 47 patients in their follow ups. It was concluded that WSS was valuable for diagnosis and intervention planning. Therefore, the increasing number of clinical studies for investigating WSS, motivated us to understand how the intervention impacts WSS.

## Limitations

This study was performed on 3 patients with COA who underwent extra-anatomical bypass grafting. We observed good agreements between the velocity fields calculated using the lattice Boltzmann method and measured using MRI in 5 COA patients in this study. The lumped-parameter part of this framework was already validated against cardiac catheterization pressure gradient measured in 85 patients^29,31,42^. Moreover, the entire patient-specific computational framework (lattice Boltzmann method and lumped parameter model) was validated against clinical Doppler echocardiography previously^31^ and in this study (in 3 patients with COA in both pre and post bypass grafting status). Future studies must consider further validation of the findings of this study as well as the computational framework in a larger population of COA patients with extra-anatomical bypass grafting. However, our results demonstrate the potential of the framework to track changes in both cardiac, and vascular states in the patients investigated in this study.

## Data Availability

The development and validation of the proposed method require the retrospective clinical data routinely measured in clinics and were transferred as the de-identified & anonymized data. The code and the optimization algorithms are available from the author upon request.
